# Understanding consumer behavior in Lebanon’s polycrisis: The role of ethnocentrism, coping ability, and socioeconomic status

**DOI:** 10.1371/journal.pone.0341265

**Published:** 2026-02-05

**Authors:** Imad Bou-Hamad

**Affiliations:** Department of Business Information and Decision Systems, Suliman S. Olayan School of Business- American University of Beirut, Beirut, Lebanon; Xuzhou University of Technology, CHINA

## Abstract

Consumer behavior during crisis has grown into an increasingly significant subject of study, as financial and social disturbances affect purchase choices and market dynamics. In Lebanon, a country experiencing a prolonged economic crisis and societal instability, businesses and policymakers face urgent challenges in maintaining consumer demand for domestic products. Drawing on the Theory of Reasoned Action and the Transactional Model of Stress and Coping, this study aims to investigate the demographic, psychological, and socioeconomic predictors of ethnocentrism and purchasing intentions for Lebanese products in this environment. A quantitative approach was adopted. Data were collected from 606 Lebanese consumers through a structured survey between March and May 2021 during the country’s financial collapse, COVID-19 pandemic, and Beirut port explosion. The findings show that ethnocentrism (β = 0.253, p < 0.001) and SES (β = 1.479, p = 0.003) directly predict purchase intentions for Lebanese products. Demographic factors influence ethnocentric tendencies, and coping ability operates more strongly among older consumers (β = −0.023, p = 0.047). Ethnocentrism partially mediates the age effect, while SES shows inconsistent mediation patterns. These insights extend psychological models of behavior by demonstrating how coping mechanisms and socioeconomic standing interact with identity-based attitudes to influence consumption during national crises. The study offers theoretical contributions relevant to crisis contexts and provides practical insights for marketers and policymakers aiming to strengthen domestic markets through targeted segmentation and culturally aligned messaging.

## Introduction

Over the past decade, Lebanon has endured a series of profound crises that have disrupted the country’s economic and social structures and significantly altered consumer behavior [1,2]. These overlapping crises included the financial collapse in 2019, the COVID-19 pandemic, and the Beirut port explosion in 2020. The 2019 recession destroyed household purchasing power and undermined trust in economic institutions [3], as evidenced by the paralysis of the banking system, currency depreciation of approximately 90%, and GDP contraction of over 35%. These difficulties were made worse by the COVID-19 pandemic, which caused lockdowns, supply chain interruptions [4], and stress on the healthcare system [5]. Then, in August 2020, the tragic Beirut port explosion, which killed over 200 people and devastated essential infrastructure, weakened the economy and exacerbated social trauma [6]. These accumulating shocks have led to what academics refer to as a “polycrisis” [7], which is a confluence of social, political, economic, and health crises that work together to increase total instability more than any one crisis could.

In such turbulent environments, understanding how individuals make purchasing decisions is essential for Lebanese businesses and policymakers seeking to support domestic markets and stabilize economic activity [8]. Domestic manufacturers face existential risks as consumers struggle to balance affordability and support local businesses due to substantial income losses and shortages [9]. For instance, despite their preference for local products, financially troubled households turn to cheaper imported alternatives, resulting in a decline in market share for Lebanese food producers [10]. Small and medium-sized enterprises, which account for more than 95% of Lebanese businesses, lack the capacity to compete on price with imports, making non-economic consumer loyalty, such as national identity, critical for survival [2]. Similar challenging trade-offs have been made by policymakers about whether to adopt preferential measures that target domestic industries or to generally promote consumer purchasing power. Relatedly, designing successful company plans and economic policies during times of crisis requires an understanding of the psychological and social factors influencing consumer choices [11]. Consumer ethnocentrism, which refers to the beliefs about the morality of purchasing domestic versus foreign items, and coping skills, which are the perceived ability to manage crisis-related stress, may have a significant impact on whether customers favor local products despite economic restrictions. In times of protracted instability, these mechanisms, along with socioeconomic resources, could determine how resilience and identity translate into consumption patterns.

Prior research has shown that buyer ethnocentrism predicts local product preference during crises, as observed in post-2008 Greece [12] and Turkey during periods of economic uncertainty [13,14]. Western research has also shown that during periods of financial stress, consumption patterns are influenced by socioeconomic resources and psychological resilience or coping abilities [15,16]. In the Middle East, specifically, researchers have explored changes in consumer behavior and the influence of cultural factors in Lebanon and Oman [17,18].

While prior research has examined ethnocentrism, coping ability, and socioeconomic resources in various crisis contexts, critical questions remain about how these mechanisms operate under extreme conditions. Most studies examine ethnocentrism where national identity is relatively unified, not in contexts like Lebanon, where sectarian fragmentation and contested national narratives complicate notions of “the nation.” Similarly, research on coping and SES typically examines contexts where different groups experience varying constraint levels, not catastrophic institutional collapse affecting all socioeconomic groups simultaneously. Finally, how demographic factors moderate these pathways under compounding polycrisis, rather than isolated shocks, remains underexplored, particularly in understudied Middle Eastern contexts where collectivist orientations, sectarian complexity, and institutional arrangements may alter Western-derived theoretical relationships

To address these gaps, this study integrates four complementary theoretical frameworks that collectively explain how identity, attitudes, psychological resources, and material constraints shape crisis-driven consumption. The Theory of Reasoned Action [19] and Social Identity Theory [20] explain attitude formation and identity-based motivations, while the Transactional Model of Stress and Coping [21] and Resource-Based Theory of Stress [22] address how psychological and material resources moderate the translation of these attitudes into behavior. Grounded in these frameworks, this study aims to investigate how ethnocentrism, demographic factors, coping ability, and socioeconomic status (SES) influence purchase intentions for Lebanese products under conditions of national stress. Specifically, the analysis is guided by the following research questions:

(1) How do ethnocentrism, coping ability, and socioeconomic status shape purchasing preferences during Lebanon’s crises?
(2) How do demographic characteristics (age, gender, education, marital status) predict ethnocentric tendencies for Lebanese products?
(3) How does coping ability mediate the relationship between demographics and consumer purchase intentions for Lebanese products?
(4) What role does SES play in mediating the effects of coping and in shaping stress-related consumer behavior during Lebanon’s crises?

Beyond addressing these questions, the paper contributes theoretically by integrating identity-based (Social Identity Theory), attitudinal (Theory of Reasoned Action), psychological (Transactional Model of Stress and Coping), and structural (Resource-Based Theory) frameworks, demonstrating the sequential operation of these systems and identifying the boundary conditions that regulate established linkages. Empirically, the study offers unique quantitative information from the understudied Middle Eastern polycrisis environment of Lebanon. Practically, it provides evidence-based recommendations for companies, marketers, and policymakers about the effectiveness of nationalistic messaging in fragmented environments, and how coping strategies influence responsiveness to crisis-framed messaging.

## Theoretical framework

The two primary theoretical frameworks around which this study is based are the Theory of Reasoned Action (TRA) [19] and the Transactional Model of Stress and Coping [21]. Together, these frameworks provide an explanation of how psychological processes, attitudes, and norms influence consumer behavior in uncertain times. Additionally, the Social Identity Theory (SIT) [20] and the Resource-Based Theory of Stress [22] offer complementary perspectives that enhance the examination of consumer ethnocentrism and socioeconomic factors in crisis contexts. Despite being frequently used in Western consumer research [15,16], these theories have not received much scholarly attention in the Middle East, especially in Lebanon, where compounding crises offer particular opportunities for testing and expanding their applicability.

Based on the Theory of Reasoned Action [19], attitudes toward the action and subjective norms influence behavioral intentions, which in turn predict actual behavior. A prominent attitudinal component in this approach is consumer ethnocentrism, which is the conviction that buying domestic goods is both morally sound and beneficial to the country’s economy [23]. In Lebanon’s multifaceted crises, such views are reinforced by communal hardships, leading to strong subjective standards that favor local unity. Therefore, TRA serves as the foundation for the argument that ethnocentrism directly predicts intentions to purchase Lebanese goods, whereas demographic traits, including age, gender, education, and marital status, have an impact on these sentiments. By identifying ethnocentrism as an attitudinal driver of intention [24], TRA serves as the foundation for the study’s examination of how identity-based attitudes influence consumer decisions during times of crisis.

The Transactional Model of Stress and Coping [21], on the other hand, focuses on the process by which people assess stressful experiences and use coping strategies to manage them. This concept is particularly relevant in the Lebanese context, where buyers face ongoing political unrest and economic hardship [1]. While socioeconomic status (SES) influences access to both psychological and material coping mechanisms, coping ability is a psychological resource that can limit how stress manifests itself in consumer behavior [25]. People with higher coping skills, for example, might use stress driven by crises to make adaptive purchasing decisions, such as purposefully buying domestic goods [26]. On the contrary, people with lower socioeconomic status might have fewer options, which would limit their capacity to act on their preferences [27]. This viewpoint supports the study’s hypotheses about how coping skills influence purchasing intentions and how socioeconomic status mediates them.

While TRA and the Stress and Coping framework serve as the fundamental theoretical foundations, two additional viewpoints supplement the investigation. Social Identity Theory [20] provides supplementary insight into why ethnocentric sentiments become more prominent during national crises by explaining how group identification exacerbates in-group favoring and out-group divergence [28]. In fact, SIT enhances knowledge of the social factors underlying local product preference in Lebanon, where identity and solidarity become more prominent when threatened. Complementary to this, the Resource-Based Theory of Stress [22] enhances the coping framework by emphasizing how people’s capacity to tolerate stress is influenced by their availability to resources, regardless of whether they are psychological, social, or financial. This lens is consistent with the study’s emphasis on SES as a structural predictor of consumer resilience.

Taken together, these theories offer a logical foundation for comprehending how consumers behave during times of crisis. TRA describes how attitudes and norms influence buying intentions, whereas the Stress and Coping theory describes how SES and psychological resilience influence adaptive reactions to uncertainty. By placing consumer behavior within larger identity and resource dynamics, SIT and Resource-Based Theory enhance this basis. Through integrating these viewpoints, it becomes clearer how identity, attitude, and structural elements work together to influence consumer behavior throughout Lebanon’s protracted polycrisis, improving theoretical rigor and contextual relevance.

## Literature review

### Lebanon’s crisis environment and consumer behavior

Lebanon continues to face one of the worst and most persistent crises in its recent trajectory [29]. As of 2025, the country is still dealing with the consequences of financial collapse, ongoing currency depreciation, governmental stalemate, and significant shortages of basic goods and services [1]. These persistent circumstances have produced a persistently uncertain environment in which scarcity, inflation, and evolving survival tactics significantly impact consumer decision-making [29].

The roots of this crisis can be traced to the events of 2019−2020, including the financial collapse of 2019, the COVID-19 pandemic, and the devastating Beirut port explosion of 2020. These shocks have significantly disrupted consumer behavior and market dynamics [30]. As such, understanding shifts in consumer behavior is crucial for marketers and policymakers striving to bolster economic resilience and support local industries. Scholars have argued that understanding consumer behavior during crises is essential for navigating uncertain markets and crafting effective interventions [31,32]. The Middle East, characterized by its socio-political instability and collectivist cultural values, offers a distinctive and underexplored context for studying consumer behavior during crises. In particular, the region’s complex political landscape, coupled with strong national identities, makes it an interesting setting for examining how consumers react during crises [17].

Research on crisis-driven consumer behavior has expanded considerably, including in Middle Eastern contexts [17,18]. Building on this foundation, the current study examines how demographic, psychological, and socioeconomic factors jointly shape purchase intentions in Lebanon’s polycrisis environment, testing whether established relationships operate similarly under conditions of extreme institutional collapse and fragmented national identity.

### Consumer behavior in normal vs. crisis context

Building on Lebanon’s crisis setting, it is critical to recognize the distinctions between stable and crisis conditions in terms of consumer decision-making. During times with comparatively stable conditions, rational and utilitarian factors usually drive consumer behavior [31]. In such cases, price sensitivity, product quality, convenience, and brand reputation are all important considerations in molding purchasing decisions [33]. Customers base their evaluation of options on maximizing utility and decreasing cost, which reflects decision-making procedures based on resource stability and predictability [31]. For example, shoppers are more inclined to value price and convenience over symbolic or identity-driven buying in non-crisis situations [34].

On the other hand, during times of crisis, identity-driven mechanisms, scarcity, and uncertainty have a significant impact on consumer behavior [35]. As people view purchasing locally produced goods as an ethical responsibility and a show of support for the domestic economy, ethnocentrism frequently takes center stage [36,37]. Decisions in these situations are influenced more by coping mechanisms, group affiliation, and a propensity to reduce risk than by cost or convenience [38]. Consumers frequently choose domestic products as a symbolic act of resilience during financial downturns and natural calamities, which strengthens in-group loyalty and supports local businesses [14,39].

In times of crisis, coping mechanisms also play a crucial role in influencing consumer behavior [38]. According to the Transactional Model of Stress and Coping, people evaluate tension and use coping strategies based on their socioeconomic and psychological resources. In the light of several studies, consumers’ adaptive behaviors during the COVID-19 pandemic included stockpiling, avoiding foreign goods, and favoring reliable local brands [31]. These actions show how resilience-oriented consuming practices, which prioritize lowering risk and bolstering control, have replaced logical utility evaluation [38].

Research on consumer behavior during crises has expanded across various contexts, including natural disasters, pandemics, and economic recessions [15,16,35,38]. However, most studies examine single-crisis scenarios in contexts where institutional infrastructure remains functional. Lebanon’s polycrisis, combining financial collapse, political instability, pandemic, and infrastructure destruction, presents distinct theoretical questions about whether psychological mechanisms identified in single-crisis or stable-institution contexts operate similarly when multiple crises compound and institutional systems fail.

This study fills this gap by combining the Stress and Coping framework with the Theory of Reasoned Action to explain consumer purchase intentions in Lebanon’s crisis environment. This adds both theoretical and empirical distinctive aspects to current theories and extends them into the particular setting of the Middle East crisis.

## Hypotheses development

### Ethnocentrism and purchase intentions

Consumer ethnocentrism, defined as the belief in the moral superiority of purchasing domestic products [23], plays a pivotal role in shaping purchasing behavior during crises [37]. Studies have consistently shown that ethnocentrism peaks during times of crisis, as consumers seek to support their local economy and preserve national identity [36]. Ethnocentric consumers, according to research, prioritize local products as an expression of solidarity and support for national economic resilience, particularly in collectivist cultures such as those found in the Middle East [37,40]. The Theory of Reasoned Action further underscores this, explaining how attitudes like ethnocentrism influence behavioral actions, such as purchase intentions [19]. Moreover, the Social Identity Theory provides additional theoretical grounding, suggesting that individuals derive a sense of self from their national identity, which can amplify ethnocentric behaviors during crises [20].

Findings from crisis-affected economies, such as Greece and Turkey, have indicated that consumers demonstrate national solidarity by purchasing domestic goods [14,41]. Additionally, studies in post-crisis contexts, such as Argentina’s economic collapse, have shown that ethnocentrism can serve as a coping mechanism, reinforcing local product preferences [39]. In consistent with these findings, given Lebanon’s socio-political and economic instability [29], we hypothesize that:

**H1:** Consumer ethnocentrism positively influences the purchase intentions for Lebanese products in the country’s crisis context.

### Demographic predictors of ethnocentrism

According to Fishbein and Ajzen [19], the Theory of Reasoned Action (TRA) holds that social factors and personal characteristics shape attitudes, which in turn impact behavioral intentions. One example of such an attitude is consumer ethnocentrism, and its strength can be strongly influenced by demographic traits [42]. Social Identity Theory (SIT) also highlights how loyalty to domestic products is reinforced by affiliation with in-groups, such as family, gender norms, or national identity [20]. Hence, demographic factors such as age, education, gender, and marital status offer a useful prism through which to view differences in ethnocentric inclinations [42], particularly during times of crisis when ties to one’s country and its citizens become increasingly significant.

Previous research has indicated that age is a strong predictor of ethnocentric attitudes, with older consumers exhibiting greater loyalty to domestic products due to more ingrained nationalistic values [42]. This is supported by generational cohort theory, which posits that older generations, having experienced formative events like wars or economic booms, develop stronger attachments to national identity [43].

Conversely, education has been shown to decrease ethnocentrism since exposure to many perspectives and globalized contexts promotes openness and universal attitudes [44]. However, the link is not always linear in weak economies; even when they have greater access to international purchasing options, highly educated people may still choose domestic goods as a symbolic gesture of solidarity or resistance [36]. Therefore, during times of crisis, education can promote global knowledge while not completely eradicating nationalistic sentiments [45].

Gender differences have also been observed, with females often displaying higher ethnocentric attitudes than males [42]. This could be related to socialization patterns that prioritize caring duties, family safety, and community welfare, all of which are consistent with bolstering domestic markets in times of uncertainty [46].

Similarly, marital status has been linked to ethnocentrism, as married individuals may be more concerned with household stability and collective identity, which reinforces national allegiance [47]. In fact, married individuals may be particularly motivated to prioritize local items in the context of Lebanon’s crisis, where households are burdened by shortages and inflation, to safeguard family welfare and support national resilience [48].

Taken collectively, these demographic traits offer significant preconditions for comprehending variance in consumer ethnocentrism [42]. They demonstrate how identity and social structure influence customer reactions in times of crisis by tying opinions toward domestic products to personal life experiences, social roles, and educational exposure [42,44,48]. These elements are particularly relevant in Lebanon’s concurrent crisis, as households deal with uncertainty and scarcity in ways that strengthen their ties to regional goods. Building on these findings, we hypothesize that:

**H2a:** Age positively influences consumer ethnocentrism in Lebanon’s crisis context.

**H2b:** Education negatively influences consumer ethnocentrism in Lebanon’s crisis context.

**H2c:** Females exhibit higher levels of consumer ethnocentrism than males in Lebanon’s crisis context.

**H2d:** Married individuals exhibit higher levels of consumer ethnocentrism than single individuals in Lebanon’s crisis context.

Older cohorts have been found to have higher ties to national identity, making age one of the most significant demographic factors impacting ethnocentric beliefs [42]. However, age’s significance extends beyond its obvious connection to ethnocentrism. Conforming to the Theory of Reasoned Action [19], predispositions are converted into behavioral intentions through attitudes, such as ethnocentrism. Relatedly, age-based interactions and generational experiences strengthen in-group loyalty, which in turn fuels supportive consumption patterns, according to SIT [20]. Ethnocentrism is a primary means of understanding how age influences buying intentions in the context of Lebanon’s crisis, where concerns about economic survival and community solidarity are paramount [49]. Hence, we hypothesize that:

**H3:** Ethnocentrism mediates the relationship between age and purchase intentions for Lebanese products in the country’s crisis context.

### Psychological factors and coping ability

To understand how consumers make purchasing decisions during emergencies, psychological factors play a crucial role [50]. In accordance with Lazarus and Folkman’s [21] Transactional Model of Stress and Coping, coping skills influence how people evaluate stress and deal with uncertainty. Coping ability serves as both a protective mechanism and a predictor of adaptive consumer behavior in situations of protracted challenges, such as the financial crisis in Lebanon and the COVID-19 pandemic [51]. The ability to cope with stress has been shown to mediate how individuals react to economic or social turmoil, affecting their purchasing behaviors [52]. In other words, higher coping skills make a person more capable of handling stress, keeping things under control, and converting views like ethnocentrism into intentional buying behaviors. On the other hand, weaker coping skills are linked to less planning, maladaptive decision-making, and increased susceptibility to outside influences, all of which might erode purchasing intentions [53].

Crucially, coping skills can also interact with demographic traits [54]. As demonstrated in earlier studies, older consumers tend to exhibit more ethnocentrism [42], yet this effect can vary depending on how well they handle stress [54]. While people with less advanced coping skills might not be able to change their views, those with greater coping skills might continue to have strong intentions to buy local goods in spite of uncertainties [55]. As a result, coping mitigates the impact of age on purchase intentions, highlighting its function as a direct predictor and an interaction term in consumer behavior driven by crises. Thus, we hypothesize that:

**H4a:** Coping ability positively influences the purchase intentions for Lebanese products in the country’s crisis context.

**H4b:** Age moderates the relationship between coping ability and purchase intentions for Lebanese products in the country’s crisis context.

### Socioeconomic status as a mediator

Socioeconomic status (SES) exerts a significant impact on consumer behavior during crises [56], given that it indicates access to financial, social, and educational resources [57]. In agreement with the Resource-Based Theory of Stress [22], people who possess more resources are better equipped to handle challenges and maintain adaptive behaviors [57]. Higher SES gives consumers more freedom to keep buying local goods in light of political unrest and economic collapse, both as a practical need and as a show of solidarity with their country [56]. Lower SES, in contrast, limits consumer choice and frequently forces people to put affordability and immediate survival ahead of identity-driven purchases [39].

Another psychological resource that influences outcomes in crises, in conjunction with SES, is coping ability [58]. Coping techniques influence how stressors are perceived and converted into behavioral reactions, in adherence to the Transactional Model of Stress and Coping [21]. Empirical research has shown that higher coping efficacy is associated with more robust consumption choices, less fear or avoidance, and a greater propensity to match purchases to long-term objectives [52,55]. Crucially, coping explains why people with comparable material constraints have different behavioral intentions and supports the adaptive use of socioeconomic resources [58]. Building on these insights, within the unique context of Lebanon’s financial and political crises, we hypothesize that:

**H5a:** SES positively influences the purchase intentions for Lebanese products in the country’s crisis context.

**H5b:** SES mediates the relationship between coping ability and purchase intentions for Lebanese products in the country’s crisis context.

Coping ability can also be viewed as a mechanism that connects socioeconomic status to stress outcomes, in addition to its role in purchase intentions [52]. Following the Resource-Based Theory of Stress [22], people who have more socioeconomic resources are less stressed because they have access to social and financial safety nets against outside shocks. However, psychological resilience plays a major role in this buffering effect [59]. Within the Transactional Model of Stress and Coping [21], stress is influenced by both structural factors and a person’s ability to recognize and handle difficulties. The argument is supported by empirical data, which indicates that coping effectiveness lessens the degree to which low SES results in increased psychological discomfort [55,60]. Therefore, we hypothesize:

**H6:** Coping ability mediates the relationship between SES and stress levels in Lebanon’s crisis context.

It is worth mentioning that stress plays a critical role in influencing consumer decisions during times of crisis [61], frequently increasing risk aversion and decreasing propensity to make discretionary or supporting purchases [31]. Although stress is not measured explicitly in this study, its significance as a result of SES and coping can be determined by its relevance in both the Resource-Based Theory of Stress and the Transactional Model of Coping. Hence, by including stress in the framework, the study broadens its theoretical contribution and emphasizes the necessity of additional Lebanese crisis-context research to confirm this pathway empirically.

**Fig 1:**
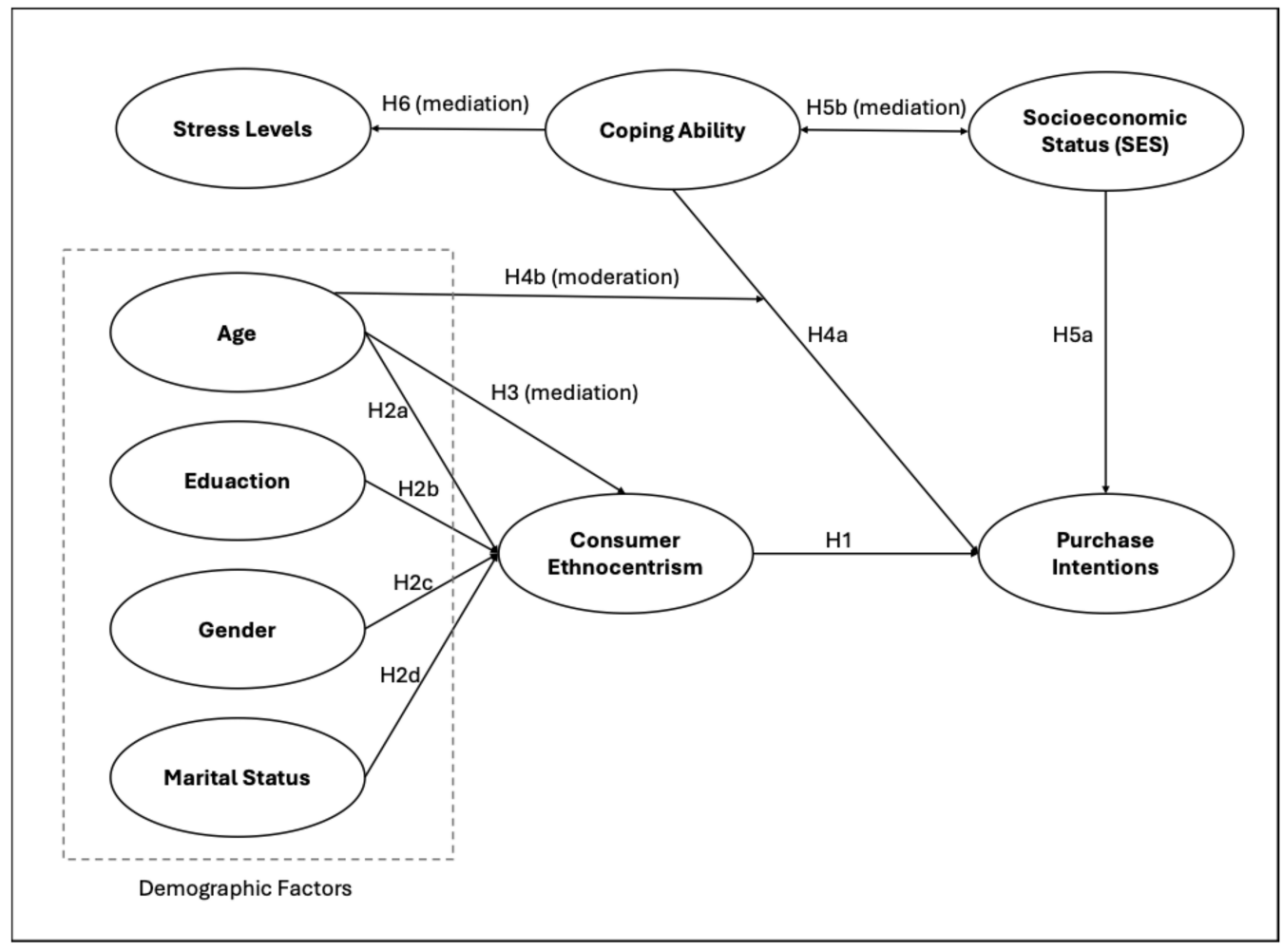
Proposed conceptual model.

In the context of Lebanon’s dual crises, Fig 1 shows the conceptual model created for this study, which demonstrates the proposed connections between demographic, psychological, and socioeconomic characteristics with buying behaviors.

## Methodology

### Sample and data collection

To investigate the demographic, psychological, and socioeconomic drivers of ethnocentrism and purchase intentions in Lebanon’s crisis setting, a quantitative method approach was adopted using a structured questionnaire.

The study employed a convenience sampling approach to collect data from Lebanese consumers. Although convenience sampling is less generalizable than probability approaches, it was thought to be suitable for the study’s theory-testing goals and the complex socioeconomic situation during Lebanon’s crisis [62]. To optimize sample diversity and minimize single-source bias present in convenience sampling, participants were recruited through multiple channels, including social media platforms (e.g., LinkedIn and WhatsApp), snowball sampling through personal and professional networks, and mall intercepts [63].

To meet the inclusion requirements, participants had to be Lebanese citizens of a minimum legal age of 18 years old and living in Lebanon at the time of data collection. These requirements made sure that responders were engaged in the local consumer market and had firsthand knowledge of Lebanon’s difficult circumstances. The final sample included 606 individuals who completed the survey in its full. The lack of quota sampling to guarantee proportional representation across demographic characteristics like age, gender, or education level is a drawback that we admit. In the results section, we display the final sample’s demographic makeup to evaluate its representativeness in relation to Lebanon’s population profile.

Using established scales from the literature on ethnocentrism and consumer behavior, the questionnaire was first created in English. Given Lebanon’s bilingual community, the survey was translated into Arabic using Brislin’s [64] back-translation technique to make it accessible to Lebanon’s multilingual populace. Specifically, the English version was translated into Arabic by a bilingual expert, and then back-translated into English by another independent expert. After that, a research team evaluated the versions to identify differences, addressing disagreements through discussion to achieve semantic equivalence while preserving natural Arabic expression.

To evaluate the questionnaire’s cultural appropriateness, translation accuracy, and clarity, a pilot study was carried out with 20 Lebanese customers (10 completing the Arabic version and 10 completing the English version). Minor phrasing changes were made in response to pilot feedback in order to increase clarity in both language versions, improve face validity, and guarantee linguistic equivalency [65].

Data were collected from March 1, 2021, to May 30, 2021, during an intense phase of the socioeconomic crisis in Lebanon. Prior to data collection, approval was obtained from the Institutional Review Board (IRB) of the American University of Beirut to ensure ethical compliance (IRB approval number: SBS-2020–0509). All procedures adhered to the ethical standards of the 1964 Declaration of Helsinki and its subsequent amendments.

For participants approached in public settings (e.g., malls), the purpose of the study was explained verbally by a trained research assistant. These participants were informed that participation was voluntary, involved minimal risk, and that their responses would remain anonymous. Those who agreed to participate gave verbal consent, which was considered valid and ethically acceptable under the approved protocol [66]. Their agreement to proceed and complete the questionnaire was taken as an indication of informed consent.

For the online survey, a written consent process was implemented. A detailed text preceded the questionnaire link, clearly explaining the study’s objectives, minimal associated risks, confidentiality measures, and the voluntary nature of participation. Participants were explicitly informed that by clicking the link and submitting the survey, they were providing their informed consent [66].

The questionnaire included mandatory responses to guarantee data completeness. Participants were randomly assigned to one of two identical questionnaires, differing only in a single scenario-based item related to purchase intention:

1. A Lebanese company producing a wafer chocolate bar at a specific price.
2. A foreign (Turkish) company producing a similar wafer chocolate bar at the same price.

Despite the fact that data collection occurred in 2021, resulting in a 4-year gap from the publishing of this research, which we acknowledge as a limitation, the findings retain relevance for two main reasons. First, Lebanon’s crisis conditions have persisted rather than resolved: the currency devaluation, banking paralysis, and institutional collapse that characterized 2021 have continued through 2025, with the Lebanese pound maintaining over 90% devaluation and essential services remaining dysfunctional. Second, and more importantly, this study’s primary contribution is theoretical rather than predictive, examining fundamental psychological mechanisms (ethnocentrism, coping, identity-based consumption) that operate across crisis contexts, not providing time-sensitive forecasts of Lebanese consumer behavior. The 2021 data capture crisis-driven psychological processes during Lebanon’s acute collapse phase, offering insights into how these mechanisms function under extreme conditions that remain applicable to understanding crisis consumption more broadly. The limitations section provides a detailed discussion of the ramifications of this temporal gap.

### Measures

To achieve the objectives of this study, measures were assessed using validated multi-item scales that demonstrated high to acceptable internal consistency, as indicated by Cronbach’s alpha values.

Purchase intention served as the primary dependent variable and was measured using a six-point semantic differential scale adapted from Dodds et al. [67] with three items assessing the likelihood of purchasing the product, willingness to buy the product, and probability of considering buying the product. The scale demonstrated high reliability, with Cronbach’s alpha values of 0.90 for the Lebanese product scenario and 0.88 for the foreign product scenario, both exceeding the recommended threshold of 0.70 [68].

Consumer ethnocentrism, which represents the tendency to favor domestically produced goods over foreign alternatives, was assessed using a seven-point Likert scale (1 = strongly disagree, 7 = strongly agree) adapted from Jiménez-Guerrero et al. [69]. The scale included six items, such as “A real Lebanese should always buy Lebanese-made products,” and demonstrated high internal consistency, with a Cronbach’s alpha of 0.84, showing high reliability.

Coping ability, reflecting a person’s capacity to adapt to difficult situations, was measured using a six-point Likert scale (1 = strongly disagree, 6 = strongly agree) adapted from Sinclair and Wallston [70]. The scale included four items, such as “I look for creative ways to alter difficult situations.” Cronbach’s alpha yielded 0.60, which is below the conventional threshold of 0.70, but is considered acceptable for exploratory research [71]. We recognize this reduced reliability as a drawback. As a result, throughout the research, data on coping ability were interpreted with proper caution because lower dependability may weaken observed connections and decrease statistical power.

Social class was assessed using the MacArthur Scale of Subjective Social Status [72], where participants ranked themselves on a 10-point ladder (1 = lowest social standing, 10 = highest social standing) to indicate perceived socioeconomic status. In addition to perceived social class, the socioeconomic status (SES) was determined using a standardized composite score that combined demographic factors such as income and education with the MacArthur Scale, providing a more objective measure of participants’ actual socioeconomic standing.

Standard demographic variables, including age (years), gender (male/female), education, marital status (single, married, divorced), and household size (number of people living in the house), were measured through self-report.

All the items adapted are represented in Table 1.

**Table 1:** Measurement scales: constructs, items, and sources.

Variable Source	Items	Definition
Purchase Intentions [67]	1. The likelihood of purchasing this product is high. 2. The probability that I would consider buying this product is high. 3. My willingness to buy the product is high.	Purchase intention refers to a consumer’s stated likelihood or willingness to buy a specific product or service in the future.
Ethnocentrism [69]	1. Lebanese people should always buy Lebanese-made products instead of imports 2. Purchasing foreign-made products is un-Lebanese. 3. A real Lebanese should always buy Lebanese-made products. 4. We should buy products made in Lebanon instead of allowing other countries to get rich at our expense. 5. It is best to always buy Lebanese products. 6. Lebanese should not buy foreign products that harm Lebanese companies and cause unemployment.	Consumer ethnocentrism is the tendency for customers to favor domestically produced items over those made abroad because of their sense of national identity and their conviction that buying foreign goods is immoral and detrimental to their nation’s economy.
Coping Ability [70]	1. I actively look for ways to replace the losses I encounter in life. 2. I believe that I can grow in positive ways by dealing with difficult situations. 3. I look for creative ways to alter difficult situations. 4. Regardless of what happens to me, I believe I can control my reaction to it.	Coping ability refers to an individual’s perceived capacity to effectively manage and adapt to stressful situations through cognitive and behavioral strategies.

### Analytical approach

To address the research questions and test the hypothesized connections, this study employed a combination of descriptive statistics, regression-based methods, and mediation analysis. All statistical analyses were conducted using the R programming language (version 4.4.0). Prior to hypothesis testing, a number of preparatory studies were carried out to evaluate the quality of the data and guarantee that the statistical processes that followed were satisfactory.

First, descriptive statistics were computed to summarize the key study variables. Next, multiple regression models were estimated to examine the predictors of ethnocentrism and purchase intentions for Lebanese products. Standard errors, p-values, regression coefficients (Beta), and 95% CIs were computed for every model. R^2^ and R^2^ adjusted values were used to evaluate model fit. Effect sizes (Cohen’s f²) were also calculated for all significant predictors to evaluate practical significance alongside statistical significance. To evaluate multicollinearity, variance inflation factors (VIF) were used. All VIF values were less than ten, indicating no substantial multicollinearity difficulties.

Mediation analyses were performed using the mediation package in R to test indirect effects following the counterfactual framework of causal inference [73]. Bootstrapped confidence intervals (5,000 replications) and predictive relevance Q^2^ were used to assess the significance of mediation effects. Additionally, interaction effects were tested to explore potential moderating relationships. This analytical strategy was chosen to test not only direct behavioral outcomes but also the psychological and structural mechanisms underlying consumer decisions in crisis contexts.

## Results

### Descriptive statistics

Descriptive statistics for the key study variables are presented in this section. Participants reported high purchase intention for Lebanese products (M = 4.185; SD = 1.245), which were notably higher than the purchase intention for foreign products (M = 3.653; SD = 1.356). Consumer ethnocentrism had a mean of 3.764 (SD = 1.146), indicating moderate ethnocentric tendencies in the population. Coping ability was moderately high, averaging 3.646 (SD = 0.575), while stress levels averaged 3.909 (SD = 1.230), showing moderate stress in the sample.

With respect to demographic characteristics, the average age of participants was 29.96 years (SD = 10.94). The sample was composed of 58.2% females and 41.8% males. Regarding marital status, 65.2% of participants were single, 30.5% were married, 1.8% were divorced, and 2.5% identified as “other.” Education level averaged 4.503 (SD = 1.976) on an 8-point scale, illustrating moderate to high educational attainment. For employment status, 53.3% of participants were employed, 29.4% were students, 11.4% were unemployed, and 5.9% reported other employment situations. The average number of children per household was 1.017 (SD = 1.435), and geographic distribution was relatively balanced, with a slightly higher percentage of participants residing in rural areas (50.5%) compared to urban areas (49.5%). Relatedly, participants’ perceived social standing on the MacArthur ladder averaged 6.328 (SD = 1.672) out of 10, demonstrating slightly above-middle subjective social class. All Descriptive statistics are presented in Table 2.

**Table 2:** Descriptive statistics of key study variables.

Variable	Mean	SD	Min	Max	N	%
**Purchasing** [Local]	4.185	1.245	1	6	304	_
**Purchasing** [Foreign]	3.653	1.356	1	6	302	_
**Ethnocentrism**	3.764	1.146	1	6	606	_
**Coping Ability**	3.646	0.575	1.5	5	606	_
**Age**	29.959	10.940	18	64	606	_
**Education**	4.503	1.976	1	8	606	_
**Children**	1.017	1.435	0	8	606	_
**SES**	6.328	1.672	1	10	606	_
**Marital Status**						
Married	_	_	_	_	185	30.5
Divorced	_	_	_	_	11	1.8
Single	_	_	_	_	395	65.2
Others	_	_	_	_	15	2.5
**Gender**						
Females	_	_	_	_	349	58.2
Males	_	_	_	_	251	41.8
**Employment Status**						
Employed	_	_	_	_	323	53.3
Unemployed	_	_	_	_	69	11.4
Student	_	_	_	_	178	29.4
Other	_	_	_	_	36	5.9
**Residence**						
Rural	_	_	_	_	306	50.5
Urban	_	_	_	_	300	49.5
**Stress Level**	3.909	1.230	1	6	606	_

These values suggest a moderate preference for Lebanese products and a moderately high level of ethnocentric attitudes among participants, providing a solid foundation for hypothesis testing.

### Hypothesis testing

Before testing the hypotheses, we checked to see if the main regression assumptions were met. Visual examination of the residual plots verified linearity and homoscedasticity, supporting the validity of the regression analyses.

Since all scales were taken from previously validated instruments that had proven to be valid and reliable in earlier studies [67,69,72], confirmatory factor analysis (CFA) and factor loadings were not performed. We relied on Cronbach’s alpha to evaluate the sample’s internal consistency.

It is also important to acknowledge that we recognize that the coping ability scale’s Cronbach’s alpha (α = 0.60) is below the standard cutoff of 0.70. Although Hair et al. [71] state that this level of reliability is appropriate for exploratory research, it implies a higher measurement error than the other scales. Considering reduced reliability may weaken observed correlations and impair statistical power, results on coping skills were evaluated cautiously.

#### H1, H4a, and H5a: Ethnocentrism, coping ability, and SES as direct predictors of purchase intentions.

The regression findings looking at factors influencing consumers’ inclinations to buy Lebanese goods are shown in Table 3. The total model explained 11.98% of the variance in purchase intentions and was statistically significant (R^²^ = 0.1198, adjusted R^²^ = 0.089, Cohen’s f² = 0.14).

**Table 3:** Regression analysis: effects of ethnocentrism, coping ability, and SES on purchase intentions for Lebanese products.

*Predictors*	*β*	*SE*	*t-value*	*95% CI*	*p*
Ethnocentrism	0.253	0.066	3.844	[0.123; 0.382]	0.000149***
Coping Ability	0.022	0.124	0.176	[-0.222; 0.266]	0.860
SES	1.479	0.492	3.006	[0.511; 2.450]	0.002877**
Gender [Male]	−0.265	0.141	−1.887	[-0.542; 0.011]	0.060
Education	−0.048	0.049	−0.971	[-0.143; 0.049]	0.332
Residence [Rural]	−0.344	0.177	−1.947	[-0.692; 0.004]	0.052
Children	−0.085	0.066	−1.282	[-0.215; 0.045]	0.201
Marital status [Married]	0.172	0.550	0.313	[-0.911; 1.256]	0.754
Marital status [Other]	−0.189	0.701	−0.269	[-1.569; 1.191]	0.788
Marital status [Single]	0.042	0.548	0.076	[-1.037; 1.121]	0.939

*β* (s) are estimated regression coefficients; *SE* are standard errors; *p* is the p-value; *95% CI* are percentile-based confidence intervals, based on 5,000 bootstrap replications

Significance codes: 0 ‘***’, 0.001 ‘**’

As hypothesized, consumer ethnocentrism was a significant positive predictor of purchase intentions for Lebanese products (β = 0.253, SE = 0.066, 95% CI [0.123, 0.382], p < 0.001), supporting H1. In particular, when all other factors were held constant, a one-unit rise in ethnocentrism was linked to a 0.259-unit increase in purchase intentions.

In contrast to H1, coping ability did not substantially predict purchase intentions for Lebanese products (β = 0.022, SE = 0.124, 95% CI [−0.222, 0.266], p = 0.860), and H4a was not supported when SES was included. This null finding implies that, rather than acting as a direct predictor, coping skills largely influence purchase intentions through indirect routes, which will be discussed in the other hypothesis.

However, purchase intentions for Lebanese products were strongly predicted by socioeconomic position, supporting H5a (β = 1.479, SE = 0.492, 95% CI [0.511, 2.450], p = 0.003). This significant positive effect suggests that throughout the crisis, customers with a better socioeconomic level have greater inclinations to buy domestic goods.

#### H4b: Age as moderator.

The regression findings examining factors influencing consumers’ inclinations to purchase Lebanese goods, including the age moderation effect, are shown in Table 4. The total model explained 11.61% of the variance in purchase intentions and was statistically significant (R^2^ = 0.1161, adjusted R^2^ = 0.083, Cohen’s f² = 0.13).

**Table 4:** Regression analysis predicting purchase intentions for the Lebanese product, with age moderation.

*Predictors*	*B*	*SE*	*95% CI*	*p*
Ethnocentrism	0.259	0.066	[0.129; 0.389]	<0.001
Coping Ability	0.742	0.359	[0.036; 1.449]	0.039
Age	0.106	0.045	[0.018; 0.193]	0.018
Gender [Male]	−0.245	0.142	[-0.523; 0.034]	0.085
Education	0.053	0.035	[-0.015; 0.122]	0.131
Residence [Rural]	−0.049	0.139	[-0.322; 0.225]	0.726
Children	0.008	0.056	[-0.102; 0.118]	0.887
Marital status [Married]	0.350	0.555	[-0.742; 1.442]	0.529
Marital status [Other]	0.027	0.713	[-1.377; 1.430]	0.970
Marital status [Single]	0.465	0.588	[-0.691; 1.622]	0.429
Coping Ability* Age (Interaction)	−0.023	0.012	[-0.046; -0.0003]	0.047

*β* (s) are estimated regression coefficients; *SE* are standard errors; *p* is the p-value; *95% CI* are percentile-based confidence intervals, based on 5,000 bootstrap replications

Coping capacity did not substantially predict purchase intentions when SES was included as a direct predictor in the previous analysis, indicating that its influence predominantly occurs through conditional or indirect pathways. However, when examining the relationship between age and coping ability without considering SES, coping ability was significantly associated with purchase intentions, supporting H4a (β = 0.742, SE = 0.359, 95% CI [0.036, 1.449], p = 0.039); yet, this effect was modest. Nevertheless, this link may be understated due to measurement error resulting from the lower reliability of the coping capacity scale (α = 0.60).

Among demographic variables, age positively predicted purchase intentions (β = 0.106, SE = 0.045, 95% CI [0.018, 0.193], p = 0.018), indicating that older customers are more likely to purchase Lebanese goods. On the other hand, gender (β = −0.245, SE = 0.142, 95% CI [−0.523, 0.034], p = 0.085), education (β = 0.053, SE = 0.035, 95% CI [−0.015, 0.122], p = 0.131), and marriage (β = 0.350, SE = 0.555, 95% CI [−0.742, 1.442], p = 0.529) did not significantly predict purchase intentions.

Critically, the interaction between coping ability and age was significant (β = −0.023, SE = 0.012, 95% CI [−0.046, −0.0003], p = 0.047), confirming H4b. This indicates that the link between coping ability and purchase intentions is moderated by age, with coping ability having a stronger effect on purchase intentions among older customers. In particular, the purchase intentions of older customers are more reliant on their perceived coping resources, whereas the intentions of younger consumers are more constant regardless of their coping capacity.

#### H2a, H2b, H2c, and H2d: Demographic predictors of ethnocentrism.

Table 5 examines the demographic predictors of consumer ethnocentrism. The overall model explained 6.25% of the variance in ethnocentrism (R² = 0.0625, adjusted R² = 0.049, Cohen’s f² = 0.07).

**Table 5:** Regression analysis: demographic predictors of consumer ethnocentrism.

*Predictors*	*β*	*SE*	*95% CI*	*p*
Education	−0.048	0.024	[-0.095; -0.002]	0.041
Age	0.006	0.006	[-0.006; 0.018]	0.340
Gender [Male]	−0.303	0.093	[-0.486; -0.119]	0.001
Residence [Rural]	0.147	0.092	[-0.033; 0.328]	0.110
Children	0.060	0.037	[-0.012; 0.132]	0.104
Marital status [Married]	0.701	0.349	[0.015; 1.387]	0.045
Marital status [Other]	0.507	0.461	[-0.398; 1.412]	0.272
Marital status [Single]	0.420	0.373	[-0.312; 1.152]	0.260

*β* (s) are estimated regression coefficients; *SE* are standard errors; *p* is the p-value; *95% CI* are percentile-based confidence intervals, based on 5,000 bootstrap replications

Consistent with H2b, education negatively predicted ethnocentrism (β = −0.048, SE = 0.024, 95% CI [−0.095, −0.002], p = 0.041), suggesting that those with greater levels of educational achievement had fewer ethnocentric inclinations. Gender had a significant effect, with males exhibiting lower ethnocentrism than females (β = −0.303, SE = 0.093, 95% CI [−0.486, −0.119], p = 0.001), supporting H2c. Marital status was also a significant predictor, as married individuals reported higher ethnocentrism scores than other groups (β = 0.701, SE = 0.349, 95% CI [0.015, 1.387], p = 0.045), confirming H2d.

Surprisingly, age in this model did not significantly predict ethnocentrism (β = 0.006, SE = 0.006, 95% CI [−0.006, 0.018], p = 0.340). Given the strong conceptual and empirical association between age and marital status, we proposed that multicollinearity might be reducing the impact of age. Hence, a secondary analysis was conducted excluding marital status (See Table 6).

**Table 6:** Regression analysis: demographic predictors of consumer ethnocentrism excluding marital status.

*Predictors*	*β*	*SE*	*95% CI*	*P*
(Intercept)	3.593	0.172	[3.254; 3.932]	<0.001
Education	−0.046	0.024	[-0.093; 0.0002]	0.051
Age	0.012	0.005	[0.003; 0.021]	0.012
Gender [Male]	−0.286	0.093	[-0.469; -0.103]	0.002
Residence [Rural]	0.139	0.092	[-0.041; 0.319]	0.130
Children	0.078	0.035	[0.008; 0.147]	0.028

*β* (s) are estimated regression coefficients; *SE* are standard errors; *p* is the p-value; *95% CI* are percentile-based confidence intervals, based on 5,000 bootstrap replications

This model explained 5.14% of variance (R² = 0.0514, adjusted R² = 0.043, Cohen’s f² = 0.054, p < 0.001). In this case, age significantly predicted ethnocentrism (β = 0.012, SE = 0.005, 95% CI [0.003, 0.021], p = 0.012), supporting H2a. This finding indicates that older consumers exhibited stronger ethnocentric beliefs during the crisis, but this relationship was partially masked in the fuller model due to overlap with marital status.

#### H3: Mediation of age’s effect on purchase intentions by ethnocentrism.

Table 7 presents the mediation analysis results for H3. The average causal mediation effect (ACME) was significant (β = 0.004, 95% CI [0.0005, 0.01], p = 0.016), indicating that ethnocentrism partially mediated the relationship between age and purchase intentions. The model showed sufficient predictive relevance (Q^2^ = 0.062), indicating that the mediation route is useful beyond sample-specific fit [74]. Specifically, older consumers tend to be more ethnocentric during a crisis, which in turn increases their intentions to purchase Lebanese products.

**Table 7:** Mediation analysis: ethnocentrism as mediator of age effects on purchase intentions.

	*Estimate*	*95% CI*	*P*
ACME	0.004	[0.0005; 0.01]	0.016
ADE	0.018	[0.003; 0.03]	0.016
Total Effect	0.022	[0.007; 0.04]	0.004
Prop. Mediated	0.166	[0.025; 0.53]	0.02

*β* (s) are estimated regression coefficients; *p* is the p-value; *95% CI* are percentile-based confidence intervals, based on 5,000 bootstrap replications

The average direct effect (ADE) remained significant (β = 0.018, 95% CI [0.003, 0.03], p = 0.016), indicating that age also affects purchase intentions through pathways other than ethnocentrism. The total effect was also significant (β = 0.022, 95% CI [0.007, 0.04], p = 0.004). Approximately 16.6% of the effect was mediated by ethnocentrism, confirming partial mediation and supporting H3 (β = 0.166, 95% CI [0.025, 0.53], p = 0.02). Although a small percentage of the age impact can be explained by ethnocentrism, the majority of the effects work through other mechanisms that were not examined in this study.

#### H5b: Mediation of coping ability’s effect on purchase intentions by socioeconomic status (SES).

Table 8 shows the mediation analysis results for H5b. The ACME was significant (β = 0.056, 95% CI [0.009, 0.120], p = 0.016), suggesting that SES partially mediates the relationship between coping ability and purchase intentions. The predictive relevance of the model was limited (Q^2^ = 0.021), just meeting the threshold for small predictive power [74]. This suggests that individuals with stronger coping ability tend to have higher SES, which in turn predicts purchase intentions.

**Table 8:** Mediation analysis: socioeconomic status as mediator of coping ability effects on purchase intentions.

	Estimate	95% CI	*P*
ACME	0.056	[0.009; 0.120]	0.016
ADE	0.1	[-0.177; 0.390]	0.448
Total Effect	0.157	[-0.122; 0.460]	0.258
Prop. Mediated	0.359	[-2.580; 3.180]	0.264

*β* (s) are estimated regression coefficients; *p* is the p-value; *95% CI* are percentile-based confidence intervals, based on 5,000 bootstrap replications

However, both the ADE (β = 0.1, 95% CI [−0.177, 0.390], p = 0.448) and the total effect were non-significant (β = 0.157, 95% CI [−0.122, 0.460], p = 0.258). The proportion mediated was 35.9% but was not statistically significant (β = 0.359, 95% CI [−2.580, 3.180], p = 0.264). According to MacKinnon et al. [75], this pattern – significant indirect effect but non-significant overall effect – indicates inconsistent mediation and points to competing pathways. Even while the SES pathway is strong and valuable, the overall link may be obscured by opposing processes or unmeasured suppressive effects. This analysis implies that the relationship between coping ability and purchasing intentions is more complex than initially thought and theoretically only weakly supports H5b. These contradictory results could potentially be caused by the coping ability scale’s lower reliability, which introduces measurement error that masks real relationships.

#### H6: Mediation of SES’s effect on stress by coping ability.

Table 9 reports the mediation analysis results for H6. The ACME was significant (β = −0.057, 95% CI [−0.130, −0.0001], p = 0.033), indicating that higher SES is associated with greater coping ability, which in turn decreases stress levels. Yet, the model showed no predictive relevance (Q² = −0.002), indicating the mediation pathway lacks practical predictive utility beyond the sample [74].

**Table 9:** Mediation analysis: coping ability as mediator of SES effects on stress.

	Estimate	95% CI	*p*
ACME	−0.057	[-0.130; -0.0001]	0.033
ADE	−0.032	[-0.441; 0.36]	0.863
Total Effect	−0.09	[-0.495; 0.30]	0.645
Prop. Mediated	0.636	[-4.215; 3.55]	0.65

*β* (s) are estimated regression coefficients; *p* is the p-value; *95% CI* are percentile-based confidence intervals, based on 5,000 bootstrap replications

Nevertheless, similar to H5b, both the ADE (β = −0.032, 95% CI [−0.441, 0.36], p = 0.863) and the total effect were also non-significant (β = −0.09, 95% CI [−0.495, 0.30], p = 0.645). The proportion mediated was 63.6% but was not statistically significant (β = 0.636, 95% CI [−4.215, 3.55], p = 0.65). This pattern again suggests inconsistent mediation in which the indirect path through coping ability is significant, but the overall link between SES and stress levels is weak or non-existent (H6) when all pathways are taken together.

Together, these results support the notion that identity-based and coping-related mechanisms influence consumer decision-making in times of national crisis, although some indirect effects remain weak or modest.

## Discussion

This study examines how consumer ethnocentrism, demographic factors, psychological resilience, and socioeconomic status (SES) jointly shape the purchase intentions for Lebanese products, particularly during Lebanon’s unique socio-political and economic crisis. The results shed light on the intricate ways that identity, life stage factors, and coping skills affect crisis-driven consumption while also exposing significant boundary constraints and surprising paths that challenge simple theoretical expectations.

***H1:*** Consistent with prior research [14,36], ethnocentrism emerged as a significant predictor of purchase intentions for Lebanese products (H1: β = 0.253, 0.066, 95% CI [0.123, 0.382], p < 0.001). This finding underscores the role of national identity and solidarity in driving consumer behavior during crises, particularly in collectivist cultures like Lebanon. The positive relationship between ethnocentrism and purchase intentions aligns with the Theory of Reasoned Action [19], which posits that attitudes strongly influence behavioral intentions. The results also echo principles from Social Identity Theory [20], where collective identity becomes more salient during periods of national threat, reinforcing in-group loyalty. This mechanism functions similarly in crises, as evidenced in Argentina’s 2001 crisis, where buying local goods represented concrete participation in national recovery efforts [76], and in post-2008 Greece, where economic collapse sparked defensive nationalism and a preference for domestic products as citizens achieved psychological control through consumption [12]. This pattern’s constancy across several crisis situations indicates that identity-based consumption is a basic psychological reaction to a shared threat that is independent of particular cultural or economic factors.

***H4a:*** There was conflicting evidence in favor of the hypothesis that coping skills directly affect purchase intentions. Coping skill showed no significant direct influence when SES was included as a covariate (β = 0.022, SE = 0.124, 95% CI [−0.222, 0.266], p = 0.860), but it did show significance when SES was removed (H4a: β = 0.742, SE = 0.359, 95% CI [0.036, 1.449], p = 0.039). This trend implies that rather than acting as a stand-alone direct predictor, coping ability’s impact on purchase intentions mainly occurs through conditional or indirect pathways. The absence of the coping effect when SES is controlled is consistent with Resource-Based Theory [22], which holds that psychological and material resources are linked. Since people with better coping skills may continue to have a greater socioeconomic position, which in turn permits intentional spending, SES may act as a mediator in the relationship between coping and purchasing behavior. On the other hand, when both resources are available, the psychological impact of coping ability may be overshadowed by the concrete impact of SES on purchasing power. Additionally, observed associations may be attenuated by the coping scale’s lower reliability (α = 0.60), especially when compared to more consistently evaluated predictors. These results suggest that rather than acting as a straightforward direct predictor, coping ability affects crisis consumption through moderated (H4b) and mediated (H5b) routes.

***H5a:*** This hypothesis was supported by the significant prediction of purchase intentions for Lebanese products by socioeconomic position (H5a: β = 1.479, SE = 0.492, 95% CI [0.511, 2.450], p = 0.003). According to the Resource-Based Theory, which holds that material resources allow for intentional, identity-aligned consumption under restrictions, this significant effect suggests that consumers with greater socioeconomic status had stronger intentions to buy domestic goods during the crisis [22]. Despite possible price premiums, higher-SES Lebanese customers have the means to prioritize domestic goods, portraying purchases as aiding in the country’s recovery rather than being pressured to choose the least expensive options. This finding is in contrast to stable economies, where extravagant spending is more accurately predicted by SES than by domestic choice. SES serves as an enabling resource in the crisis situation in Lebanon, allowing consumers to act on coping mechanisms and ethnocentric views, converting psychological preferences into behavioral intentions. The significant effect implies that SES functions as a crucial border condition; when affordability concerns predominate, even high ethnocentrism or coping skills may not translate into behavior in the absence of sufficient material means. This direct effect indicates that SES serves as both an autonomous facilitator and a mediating mechanism, complementing the indirect channel through coping ability (H5b).

***H2b:*** Ethnocentrism’s demographic predictors shed more light on how identity-based views are shaped by socialization, life stage transitions, and social embeddedness [46,48]. The study revealed that education is negatively linked to ethnocentric behaviors (H2b: β = −0.048, SE = 0.024, 95% CI [−0.095, −0.002], p = 0.041), reflecting exposure to a variety of viewpoints that stimulate global identity in competition with national identity. However, this effect is not very strong because, even among educated consumers, crisis situations trigger survival concerns and impulses for solidarity that override cosmopolitan orientations. This pattern matches the findings of Meeusen et al. [44] in the Netherlands, where individual-level traits were overshadowed by pragmatic restrictions during economic collapse, reducing the liberalizing influence of education.

***H2c:*** Gender also turned out to be a significant predictor, with women exhibiting more ethnocentrism than men (H2c: β = −0.303, SE = 0.093, 95% CI [−0.486, −0.119], p = 0.001). This gender disparity is a reflection of the different roles that women play in Lebanese culture, where they are largely in charge of taking care of the home and the family. As a result, they are more inclined to choose domestic products in order to safeguard the welfare of the family [77]. This stands in stark contrast to Western environments, where gender variations in ethnocentrism are usually nonexistent [78]. That suggests that gender matters for consumption only in cultures where the social duties and obligations of men and women are clearly distinct.

***H2d:*** Ethnocentrism was also strongly influenced by marital status, with married people exhibiting greater preferences for domestic goods than divorced and single people (H2d: β = 0.701, SE = 0.349, 95% CI [0.015, 1.387], p = 0.045). By integrating individuals into extended family networks that establish expectations to promote the common good, such as purchasing locally produced goods, marriage radically alters the social environment of consumers [19]. In alignment with the Theory of Reasoned Action, national economic stability becomes more personally meaningful for married people, especially those with children, who also change their priorities from personal preferences to family welfare [47]. In collectivist societies like Lebanon, where family responsibilities have a significant impact on everyday choices and consumption patterns, this effect is particularly noticeable.

***H2a:*** Age displayed a more nuanced pattern that only became important when the research was conducted without taking marital status into account (H2a: β = 0.012, SE = 0.005, 95% CI [0.003, 0.021], p = 0.012). This implies that life conditions that usually accompany aging, including marriage and family duties, are more important than age itself. Due to their experiences during the 1975–1990 civil war, which influenced their enduring nationalist views [79], older Lebanese consumers probably display greater ethnocentrism. Furthermore, becoming older inherently results in a stronger bond with stability and tradition [42]. Similar trends were seen in the U.S. after the war, where older war-torn generations exhibited significantly higher levels of ethnocentrism than younger cohorts [80]. This implies that experiencing national trauma results in long-lasting preferences for domestic goods that last for many years.

***H3:*** The association between age and purchase intentions was partially mediated by ethnocentrism (H3: β = 0.166, 95% CI [0.025, 0.53], p = 0.02). The idea that identity-based attitudes convert demographic traits into behavioral choices is supported by Social Identity Theory [20]. Through the cognitive appraisal process described by the Theory of Reasoned Action, purchase intentions are shaped by the stronger ethnocentric attitudes of older consumers [19]. Ethnocentrism is merely one of multiple pathways, as evidenced by the low proportion mediated. The substantial direct effect implies that age also affects intentions through different mechanisms that function independently of national identification, such as habit development, risk aversion, or accumulated experience with local brands. Identity is important, but it coexists with other age-related factors that should be investigated further, according to this partial mediation pattern.

***H4b:*** As noted previously, consumers’ intentions to buy Lebanese items were highly influenced by their perceived ability to cope with stress connected to crises, noted as their coping ability. However, the nature of this association varied depending on the age group. The significant interaction showed that older individuals were more affected by psychological resilience than younger consumers (H4b: β = −0.023, SE = 0.012, 95% CI [−0.046, −0.0003], p = 0.047). Coping skill was a substantial predictor of purchasing intentions among older persons [81]; those who felt prepared to manage the crisis exhibited strong preferences for Lebanese goods. This pattern implies that older Lebanese have evolved psychological frameworks where perceived coping capacity immediately translates into purposeful, crisis-appropriate consumption, as a result of navigating the civil war and multiple crises. Older customers actively contribute to the revival of the country by supporting domestic items when they believe they can handle stress. These obvious connections between psychological resilience and consumerism as a coping strategy may not yet be formed by younger generations, who have not had similar crisis experiences [81]. This finding is consistent with the Transactional Model of Stress and Coping [21], which emphasizes the role of coping mechanisms in managing stress and shaping behavior.

***H5b:*** A theoretically perplexing pattern emerged from the proposition that the association between coping ability and purchasing intentions is mediated by socioeconomic status. Although the indirect pathway, stronger coping ability was associated with higher SES, which in turn predicted buy intentions, was significant (β = 0.056, 95% CI [0.009, 0.120], p = 0.016), the overall relationship between coping ability and purchase intentions was not (H5b: β = 0.157, 95% CI [−0.122, 0.460], p = 0.258). According to this inconsistent mediation, the overall association is neutralized by unmeasured opposing forces even when the proposed mechanism functions as theorized. A number of explanations are worth taking into account. First, buying intentions may be influenced by higher SES through mutually reinforcing competitive pathways [82]. Higher SES may concurrently give access to imported goods, lessen emotional reliance on national identity symbols, or foster cosmopolitan consumption aspirations that favor foreign products, even though having more money makes it possible to buy domestic goods (the hypothesized positive pathway [83]. Together with a null overall effect, these conflicting mechanisms may have a sizable indirect effect. Second, even having a high socioeconomic status offers no practical benefit in the dire situation in Lebanon, where banking systems have frozen assets and currency has lost over 90% of its value [84]. Third, a more accurate assessment might reveal a bigger overall effect, but the coping capacity scale’s limited reliability introduces measurement error that blocks actual correlations.

***H6:*** Similarly, there were significant indirect effects (β = −0.057, 95% CI [−0.130, −0.0001], p = 0.033) but non-significant total effects for the hypothesis that coping ability mediates the association between stress and SES (H6: β = −0.09, 95% CI [−0.495, 0.30], p = 0.645). When all pathways were taken into account, SES did not reveal any overall link with stress, but higher SES predicted improved coping abilities, which in turn reduced stress levels. Once more, this pattern points to inconsistent mediation in which the proposed mechanism is present but faces competition from unmeasured processes. This result calls into question simple implementations of Resource-Based Theory in situations involving severe crises. According to the hypothesis, socioeconomic advantages should act as a stress-reduction mechanism by offering psychological and material resources [22]. Richer Lebanese do report having higher coping skills, which lowers stress, which is confirmed by the major indirect pathway [85]. The null overall impact, however, raises the possibility that socioeconomic advantages in Lebanon could also be accompanied by particular stressors that counteract them. People with higher socioeconomic status could suffer larger losses when their assets lose value, deal with more complicated financial issues like frozen bank accounts or feel more socially responsible and ashamed about the collapse of their country. On the other hand, even wealthy people are unable to avoid the environmental stressors that permeate daily life, regardless of personal resources, when crisis intensity reaches catastrophic levels that affect all citizens, such as fuel shortages, electrical blackouts, medical supply shortages, or political paralysis.

It is worth noting that an additional analysis examined the association between ethnocentrism and purchase intentions for a foreign (Turkish) product. While the coefficient was negative, indicating the expected inverse relationship, the result was not statistically significant (p > 0.05). This suggests that although ethnocentric consumers may prefer local products, their avoidance of foreign alternatives may not be as strong or consistent in this context. This finding may indicate a context-specific ceiling effect in ethnocentrism’s behavioral expression, where preference for domestic goods does not always equate to active rejection of foreign ones. Future research could explore whether different foreign product categories elicit stronger ethnocentric reactions. For instance, symbolic versus utilitarian products may vary in their capacity to trigger ethnocentric resistance.

Contextual specificity is highlighted by contrasting these results with consumer studies conducted outside of crises. In stable economies, socioeconomic position consistently predicts consumption, demographic effects are still minor [36], and ethnocentrism functions moderately and is subordinated to quality and price considerations. These dynamics are profoundly altered by crisis, identity becomes more important, demographic life-stage characteristics become more significant, and ironically, economic resources become less significant as systemic failures reduce the behavioral effects of stratification. Given that mechanisms functioning in one setting may weaken, reverse, or vanish in another, this pattern implies that consumer behavior theory needs specific crisis parameters to differentiate between mild economic instability and catastrophic collapse.

Table 10 clearly represents the hypothesis testing results.

**Table 10:** Hypothesis description and support verification.

Hypothesis	Description	Support
H1	Ethnocentrism positively predicts purchase intentions for Lebanese products	Supported
H2a	Age positively predicts ethnocentrism	Supported*
H2b	Education negatively predicts ethnocentrism	Supported
H2c	Gender predicts ethnocentrism (males lower than females)	Supported
H2d	Marital status predicts ethnocentrism (married higher than divorced and single)	Supported
H3	Ethnocentrism mediates the relationship between age and purchase intentions	Supported (partial mediation)
H4a	Coping ability positively predicts purchase intentions for Lebanese products	Mixed Support**
H4b	Age moderates the relationship between coping ability and purchase intentions	Supported
H5a	SES positively predicts purchase intentions for Lebanese products	Supported
H5b	SES mediates the relationship between coping ability and purchase intentions	Weak Support (inconsistent mediation)***
H6	Coping ability mediates the relationship between SES and stress	Weak Support (inconsistent mediation)***

* H2a was supported only when marital status was excluded from the model due to multicollinearity.

** H4a showed mixed support: significant when SES was excluded but non-significant when SES was included, suggesting conditional or indirect pathways.

*** *Significant indirect effects (ACME) but non-significant total effects, indicating inconsistent mediation where the hypothesized mechanism operates but does not translate into substantial overall effects.*

## Implications

### Theoretical implications

This research contributes to the theoretical literature on consumer behavior under crisis stress by specifying how the Theory of Reasoned Action [19], Transactional Model of Stress and Coping [21], Social Identity Theory [20], and Resource-Based Theory of Stress [22] interrelate in crisis contexts, exposing new boundary conditions and procedures as opposed to just using established frameworks.

The study uses a sequential causal mechanism to illustrate theoretical integration. The first process is explained by Social Identity Theory: challenges triggered by crises promote ethnocentric views as a psychological defense by activating national identity. The next phase is then explained by the Theory of Reasoned Action: these identity-based attitudes that have been aroused are converted into purchasing intentions by means of cognitive appraisal processes. The Transactional Model of Stress and Coping helps by showing that psychological resources, particularly coping ability, which allows consumers to channel identity into intentional crisis-appropriate conduct, limit this attitude-intention translation. The structural boundary is finally made clear by Resource-Based Theory: this complete psychological pathway only functions when behavioral agency is permitted by institutional functioning and material resources. Identity activation, intention formation, psychological moderation, and structural constraint are the different nodes in the causal chain that each theory tackles. This leads to true integration, where each framework explains what the others cannot contribute to.

Three new theoretical improvements are shown by this integration. First, the relationship between attitude and intention in TRA is contextual rather than universal. In contrast to stronger effects in unified societies, ethnocentrism’s modest effect in Lebanon’s fragmented context shows that identity-based attitudes translate into intentions most effectively when in-group boundaries are clear, crises are attributed to outside forces, and collective identity is cohesive. Second, the Transactional Model necessitates age-dependent specification: for seasoned consumers with interpretive frameworks for directing psychological capacity into action, coping resources are the strongest predictors of adaptive behavior. Third, there is a crucial boundary condition for Resource-Based Theory; when the intensity of a crisis reaches a point where institutional collapse (banking failures, currency destruction, supply disruptions) overcomes individual benefits, resource-behavior linkages break down. Resource theories created in stable Western contexts do not transfer to catastrophic crises where individual-level resources are neutralized by systemic failures, as this threshold effect shows.

These implications underscore the significance of cultural and political context in shaping consumer psychology, particularly in underrepresented regions like the Middle East. In doing so, it calls for more culturally sensitive models that incorporate the influence of national identity, perceived economic threat, and resource access on consumer choices. These theoretical insights may inform future research on consumer resilience, identity expression, and decision-making under uncertainty, especially in emerging or volatile markets.

### Practical implications

The findings offer several actionable implications for both marketers and policymakers operating in crisis-affected environments, weighing suggestions based on construct dependability and empirical strength. These observations are specifically relevant to Lebanon, but they also provide generalizable lessons for other crisis-affected markets dealing with comparable issues of economic instability, institutional breakdown, and identity fragmentation.

The considerable impact of ethnocentrism on purchase intentions suggests that identity-based messaging that emphasizes collective resilience and national cohesion should be given priority by marketers. By using phrases like “Building Lebanon Together” or “Your Choice Saves Our Nation,” the Lebanese industries (e.g., food and beverage, clothing) might effectively connect consumption to the country’s recovery. To elicit ethnocentric reactions at the point of sale, packaging should prominently feature Lebanese origin symbols, such as cedar trees, national colors, or “Made in Lebanon” labels.

The high-ethnocentrism segments found in this study should be the focus of demographic segmentation. Marketing efforts should distribute resources differently for married consumers, female consumers, and elderly consumers. Advertising in platforms aimed at women over 40, community centers frequented by married couples, and family-oriented media outlets, for instance, would generate more returns than general, undifferentiated campaigns. Marketers need to understand, though, that even powerful predictors only partially explained variance, suggesting that identity-based appeals should be used in conjunction with rather than in substitute of more conventional marketing elements like availability, price, and quality.

Although older customers’ purchase intentions were strongly predicted by coping capacity, caution is necessary due to the scale’s limited overall effects and modest reliability (α = 0.60). Marketers may test messages like “Take Control: Choose Lebanese” that frame domestic consumption as proactive crisis management, but they should do pilot testing because of measurement limitations. Identity-based messaging backed by more convincing empirical data should take precedence over these appeals.

Traditional socioeconomic advantages offer little protection amid catastrophic institutional breakdown, as seen by the modest SES mediation effects. Hence, from a policy perspective, Systemic reforms, such as currency stabilization, electricity provision, and banking sector reorganization, must take precedence over individual-level measures in the eyes of policymakers. Recommendations tailored to Lebanon include establishing currency exchange systems that facilitate equitable domestic transactions; offering subsidized credit lines to local companies so they can maintain low prices; and enacting preferential procurement laws that mandate government contracts be sourced domestically whenever possible.

More broadly, the results highlight the importance of investing in public initiatives that promote psychological resilience, especially among economically vulnerable populations. Educational and community-based programs that foster adaptive coping mechanisms could indirectly support consumer confidence and stabilize purchasing behavior. Additionally, efforts to reduce socioeconomic inequalities may have long-term effects on market participation and consumer loyalty to local industries.

Overall, aligning communication, segmentation, and policy efforts with psychological and identity-based drivers can strengthen consumer engagement with domestic markets during crises.

### Limitations and future research

While this study provides a novel contribution to consumer behavior literature in the Lebanese crisis context, it has several limitations that warrant consideration. First, data collection took place between March and May of 2021, resulting in a four-year gap towards publication. Although the conditions of Lebanon’s crisis (economic collapse, currency devaluation, and institutional dysfunction) have not been fixed, certain consumer behaviors might have changed. The results are only able to capture consumer psychology during the acute crisis period because of this temporal difference. However, rather than making predictions right away, the study’s main contribution is theoretical, showing the ways in which identity, demography, and psychological resources influence crisis consumption. Longitudinal studies monitoring the evolution of these linkages as crises move from acute to chronic phases should be the focus of future research.

Second, convenience sampling was employed without quota controls, which limits generalizability. While accepted in exploratory research, certain demographic segments may be under- or over-represented [86,87]. Hence, future research may benefit from employing probability or stratified sampling techniques to strengthen external validity.

Third, the study’s cross-sectional design restricts causal inference, as it captures a single point in time. Longitudinal or experimental designs could better elucidate the dynamic relationships among ethnocentrism, psychological resilience, and purchase intentions during ongoing or repeated crises. Such approaches could also clarify the directionality of the coping–behavior link.

Fourth, self-reported responses are also subject to potential biases, particularly in assessing identity-related constructs like ethnocentrism or psychological stress. Future research could incorporate behavioral measures (actual purchase data), implicit association tests, or multi-source data to triangulate and validate the findings.

Furthermore, we relied on Cronbach’s alpha and did not disclose individual factor loadings for the measurement scales or perform confirmatory factor analysis (CFA). CFA would have offered further assurance of construct validity in the Lebanese crisis context, even though all scales were taken from previously validated instruments with proven reliability and validity in earlier research [67,69,70,72]. Thus, to increase trust in construct measurement, future studies should validate the whole measurement model.

Another significant methodological consideration involves the coping ability scale, which showed moderate reliability (Cronbach’s alpha = 0.60). While suitable for exploratory research, future studies should adopt or develop more psychometrically robust tools to improve the measurement of psychological resilience. Combining self-report items with behavioral or observational data may enhance construct validity.

While an additional analysis examined consumer reactions to a foreign (Turkish) product, the results were not statistically significant. Future work could investigate how specific product categories, perceived threats to national identity, or brand origin shape ethnocentric avoidance behaviors. This could deepen our understanding of when and why consumers selectively reject foreign alternatives in crisis contexts.

Finally, transferability of findings is restricted by Lebanon’s distinct mix of cosmopolitan heritage, sectarian disintegration, and disastrous institutional breakdown. It is unknown if similar tendencies appear in cohesive civilizations or crises of varying intensities. To distinguish between mechanisms that are universal and those that are context-specific, future research should carry out cross-national comparative studies across a variety of crisis situations, such as other Middle Eastern countries (Syria, Yemen), post-conflict Balkans (Croatia, Bosnia), or Latin American economic crises (Argentina, Venezuela).

## Conclusion

This study provides a comprehensive examination of the factors influencing purchase intentions for Lebanese products during crises. The findings highlight the critical roles of ethnocentrism, demographics, psychological resilience, and socioeconomic status in shaping consumer behavior. The study’s theoretical and practical contributions underscore the importance of considering local context and cultural dynamics in understanding consumer behavior.

By integrating these variables into a unified framework, the research advances theoretical understanding by demonstrating how psychological and structural factors jointly drive consumer decision-making under stress, extending established frameworks such as the Theory of Reasoned Action and the Transactional Model of Stress and Coping. It also responds to a broader call for culturally grounded consumer research by situating these mechanisms in a Middle Eastern context that remains underrepresented in the literature.

The study also offers valuable insights for marketers and policymakers aiming to promote local products and support economic resilience in crisis-affected regions. This integrative perspective is particularly relevant for contexts characterized by socio-political instability and national identity salience.

Future research could build on these findings by exploring additional predictors of purchase intentions, such as cultural values, social norms, and economic policies, in other crisis-affected regions. Longitudinal or experimental studies could also shed light on the evolving role of identity and resilience in shaping behavioral adaptation to prolonged instability. Ultimately, this research contributes to a growing body of work emphasizing the psychological and societal foundations of consumption in uncertain times.
